# Physiological and Therapeutical Action of Cod-Liver Oil

**Published:** 1867-05

**Authors:** A. Bissell

**Affiliations:** New York


### PHYSIOLOGICAL AND THERAPEUTICAL ACTION OF COD-LIVER OIL.

By A. BISSELL, M.D., of New York.

The discussion of the therapeutical applications of cod-liver oil, started by Dr. Joseph Adolphus, of Hastings, Mich., in his able communication in the *Reporter* of December 8th, 1866, has led me to offer the following remarks on the subject for your columns.

M. Bouchardat, Professor of Hygiene at the Academy of Medicine, Paris, says:

"The minute division of the iodine in cod-liver oil, the particular state in which it exists, must singularly facilitate its absorption by the tissues, and can in this way contribute more than the absolute proportion of this substance to the marked effects which this oil exerts on the animal economy.

"Also, iodine in the oil is not eliminated from the system, AS THE OTHER SOLUBLE PREPARATIONS OF IODINE: in this elementary combination its action is slower, more regular, and more persistent, as it is successively set at liberty in the economy, in proportion as cod-liver oil is gradually decomposed in the blood."—[*Manuel de Materie Medicale, page 749.—1856.*]

The action of cod-liver oil on the system is a double one; it is nourishing by its fatty elements, and curative by its medicinal bodies—iodine, bromine, and phosphorus, which it naturally contains; and to these three substances must be attributed its superiority over other fats or oils, either animal or vegetable, in the cure of diseases. These facts, discovered and proven by physiologists in their experiments on animals, and confirmed by the experience of physicians in their daily practice, have been corroborated during the last eight years, in a most illustrative manner, by the administration, to a large number of patients, of cod-liver oil five times richer in iodine, bromine, and phosphorus than any of the cod-liver oils known before.

Cod-liver oil, as well as other fatty substances, when taken in too large quantities, is apt to disturb the stomach and derange the functions of the intestinal canal. Only a small quantity can be digested and assimilated, the rest passing oft unchanged, producing more or less frequent and abundant alvine evacuations, in which are contained the superfluous oils or fats. Observations prove that the gastric juice has no action whatever on fats or oils, the pancreatic juice being the only body, which, by its emulsive properties, causes the absorption of these substances into the economy; and, therefore, all the oil not emulsioned by the pancreatic juice is evacuated by the intestines just as it was taken. The knowledge of this important fact is due to the recent observations of Claude Bernard, a well known authority in physiology. The oil once emulsioned by the action of the pancreatic juice, is brought into the general current of the circulation as follows: it is first taken up by the chyliferous vessels on the surface of the small intestines, and passing through the mesenteric glands and the thoracic duct, it is discharged in the left subclavian vein, where it mingles with the venous blood returning to the right cavities of the heart. This blood, and the fresh nutritious elements, furnished by the two subclavian veins, are pressed into the lungs to be there oxidized and altered; while passing through the pulmonary circulation, the oily molecules are modified and almost all of them destroyed. The blood, then ready anew for nutrition, passes into the left ventricle, to be thence distributed through the arterial system, carrying along with it, some oily globules left undecomposed during their speedy passage through the lungs, said oily globules to be successively altered in the circulating blood.

The medicinal oil, evidently brought undecomposed into the lungs, and partly in the general current of the circulation, is modified, losing not only its emulsive form, but also its oleaginous characteristics, so as to constitute a part of the arterial blood. Iodine, bromine, and phosphorus, are then set free during the process of nutrition of the tissues, each part of our system appropriating to itself the substance it needs.

The tissues, in contact with the nutritious blood, having a tendency to appropriate to themselves the elements most proper to maintain their healthy condition or to alter it, when unhealthy, is it not judicious to conclude that the lungs first and then the rest of the system, when affected with bronchitis, phthisis, scrofula, under any variety, or rickets, etc., etc., are highly benefited by the healing and restorative action of the oil and its medicinal constituents, minutely, naturally, and persistently brought into contact with the diseased parts?

That oils and fats are successively carried through the economy, and transformed, as above described, is amply demonstrated by the experiments of the most eminent modern physiologists, such as Claude Bernard, Tiedemann and Gmelin, Leuret and Lassaigne, Sandras, Bouchardat, Blondlot, Delafond, Gruby L. Corvisart, J. C. Dalton, jr., A. Flint, R. Dunglison, etc.

We must not forget this important point, that oils or fats go into the blood undecomposed and unchanged, being merely infinitesimally divided by the pancreatic juice; but if an oil contains substances, in a close chemical combination, so that they cannot be easily separated, these substances will of course be carried into the blood with the oil itself. This is just the case with a cod-liver oil which contains a large proportion of IODINE, BROMINE, AND PHOSPHORUS. Iodine and bromine have so strong an affinity for oil, that they cannot be separated from it by chemical reagents, not even by strong sulphuric acid. They must, therefore, be carried with the blood and liberated when the oil is transformed, in the process of nutrition, into its elements, and becomes the chief agent by which the heat of the body is maintained. Knowing, then, that to the nutritive property of the oil is superadded the alterative, and stimulating power of a comparatively large quantity of iodine, bromine, and phosphorus, who can doubt the efficacy, as a medicine, of cod-liver oil, if made richer with these substances?

Phosphorus, a part of our brain and bones, is a powerful diffusible stimulant, exciting the nervous organs, heightening the muscular power and mental activity, and relieving the despondency of mind occasioned by many serious diseases.

Iodine and bromine are superior to all alteratives for improving and purifying the depraved nature of the blood. They are the best remedies we possess for checking and controlling the swelling and induration of the glandular system, the ulcerative process in scrofulous complaints, the diseases of the lungs, etc. Obviously, the main point, in such serious affections, is to check and control at once the ulcerative process, and to do so it is of the greatest importance to use PROMPT AND ACTIVE MEDICATION.

SUPERIORITY OF IODINIZED COD-LIVER OIL OVER SIMPLE COD-LIVER OIL.

Until of late, natural and pure cod-liver oil has been the best remedy, and the one most generally used, with more or less success, in diseases of the lungs when of a tuberculous character. The period of the malady when the oil was first employed, and also the purity and strength of the remedy accounting for the success or failure.

Pure cod-liver oil is more likely to cure consumption, scrofula, rickets, swelling of the glands, etc., in the first stage of the disease; in the second and third stages it mitigates the severity of the symptoms and prolongs the life of the patient, but seldom saves it.

The reason for this difference of action is simply that the pure oil contains iodine, bromine, and phosphorus only in minute quantities, which although sufficient to cure a disease in the beginning, is not powerful enough when it assumes a graver type.

If we suppose for an instant the discovery of a new natural cod-liver oil, containing more iodine, bromine, and phosphorus than the oil in present use, there is not the least doubt but that every physician would prescribe it in preference, fully confident of its enhanced qualities. The natural consequence of this proposition explains satisfactorily why the medical profession should give the preference to iodinized cod-liver oil, which contains a larger proportion of iodine, bromine, and phosphorus than the oil in present use; these active elements, as before remarked, are in such a peculiar combination that their action is slow, regular, and persistent, being successively set at liberty in the economy in proportion as the oil is decomposed in the process of animal life.

Some physicians are so well convinced that the curative properties of the oil reside in these three substances, that to obtain a full effect they prescribe very large doses of the oil, sometimes giving two, three, and even four tablespoonfuls three or four times a day, the larger quantity amounting to no less than half a pint daily. That their object is not attained is fully proven by physiologists, who have demonstrated that only the small quantity of oil emulsionized by the pancreatic juice is digested and carried into the blood, the rest being passed off nearly as taken.

In view of the above physiological and chemical facts, experiments were made in 1858, which, after many trials, succeeded in preparing a *compound idinized cod-liver oil*, which is simply the best Newfoundland cod-liver oil combined with four times as much of iodine, bromine, and phosphorus as that naturally contains.

Pure cod-liver oil varies considerably in composition, as may be seen by comparing the different analyses published in works of chemistry and materia medica. A quart contains 1 to 4 grains of iodine; $\frac{1}{8}$ to $\frac{3}{4}$ of a grain of bromine; $\frac{1}{4}$ to $\frac{1}{2}$ of a grain of phosphorus. In 1860, there was published in the *Repertoire de Pharmacie*, edited by Professor BOUCHARDAT, at Paris, the formula of a cod-liver oil, which contains per quart in addition to the above quantities

| | |
|---|---|
| Iodine, | 16 grains. |
| Bromine, | 2 grains. |
| Phosphorus | 2 grains. |

The combination is made so that the oder, taste, and color of the natural oil are preserved.

This preparation being consequently five times more active than the richest commercial cod-liver oil, will tend to restore health by its curative action thus enhanced, in a much shorter time than the simple kind, and attain the desired effect where the other will fail.

The dose of this oil is *only* a tablespoonful for adults, and a dessert or a teaspoonful for children, according to age, three times daily. It may be administered at any hour, but it is preferable to select the time of meals, since we know that the pancreatic secretion manifests itself only during the stomachal digestion, to act immediately on the alimentary principles as soon as they pass from the stomach into the intestines. Though the quantity of iodine is very small in each dose, it acts nevertheless with a greater efficacy than a larger quantity of any of the iodides, for the reason stated by Professor BOUCHARDAT and others, that iodine in cod-liver oil is not eliminated from the system as the other soluable preparations of iodine, but is successively deposited in the economy as the oil is gradually decomposed in the blood.

*When iron is required with the oil, the dragees or syrup of pyrophosphate of iron will be found the most agreeable and active adjuvant. It is best for children and delicate persons to take the syrup of iron after the oil."*—[*Medical and Surgical Reporter.*

